# Psychological Intervention for Depression and Anxiety in Hemodialysis Patients: A Meta-Analysis

**DOI:** 10.62641/aep.v53i1.1628

**Published:** 2025-01-05

**Authors:** Siming Yan, Xueli Zhu, Zhongcui Huo, Zhiying Wang, Huifen Cui

**Affiliations:** ^1^Blood Purification Center, First Affiliated Hospital of Huzhou Normal College, 313000 Huzhou, Zhejiang, China

**Keywords:** psychological intervention, hemodialysis, depression, anxiety, living quality

## Abstract

**Background:**

With the advancement of blood purification technology, there is increasing attention to the mental health of hemodialysis patients, particularly concerning depression. This study aims to determine the effect of psychological interventions on anxiety and depression in hemodialysis patients through a meta-analysis.

**Methods:**

A computerized search was conducted to identify randomized controlled trial (RCT) studies published in PubMed, Embase, Web of Science, ScienceDirect, and Cochrane Library databases from their inception to October 2023, focusing on the effects of psychological interventions on improving depression in hemodialysis patients. Data extraction, quality evaluation, and cross-checking were performed independently by two researchers. The methodological quality of the included studies was assessed according to the criteria recommended by the Cochrane Handbook for Systematic Reviews and the meta-analysis was performed using RevMan 5.4 software (The Nordic Cochrane Centre, Copenhagen, Denmark). The effect of psychological interventions on anxiety and depression in hemodialysis patients was determined by combining effect sizes and I^2^ statistics.

**Results:**

Fifteen studies were included, encompassing a total of 929 hemodialysis patients: 468 in the intervention group and 461 in the control group. The results indicated that psychological interventions could improve depressive moods [mean difference (MD) = –4.91, 95% confidence intervals (CI) (–6.56, –3.26), *p* < 0.001] and anxiety status [MD = –5.11, 95% CI (–6.97, –3.25), *p* < 0.001]. A subgroup analysis based on the intervention duration (more or less than 8 weeks) revealed that patients experienced significant improvements in depression and anxiety regardless of the intervention length. Additionally, subgroup analyses focusing on quality of life demonstrated that psychological interventions significantly improved the psychological aspects of patients' quality of life [MD = 7.31, 95% CI (1.06, 13.56), *p* = 0.001]. Sensitivity analysis, which excluded sources of heterogeneity, indicated that psychological interventions significantly enhanced both the psychological [odds ratios (OR) = 4.14, 95% CI (1.08, 7.20), *p* = 0.008] and physical [MD = 2.52, 95% CI (0.10, 4.95), *p* = 0.04] aspects of patients' quality of life.

**Conclusion:**

Psychological interventions can significantly alleviate depression and anxiety in hemodialysis patients and improve their quality of life. Psychotherapy holds promise as an effective method for improving depression in dialysis patients.

## Introduction

With economic and societal development, as well as the aging population, the number of people suffering from chronic diseases has been increasing [1]. Chronic kidney disease (CKD) has become one of the primary common health concerns globally [2]. In 2012, the results of China’s first multicenter survey on the epidemiology of CKD demonstrated that the total prevalence of CKD in China was 10.8% [3]. There are nearly 119.5 million CKD patients in China [4]. The latest statistics from 2017 showed that there were 520,000 dialysis patients in China, including 450,000 hemodialysis patients [5]. The main therapy for end-stage renal disease (ESRD) in clinical practice is renal replacement therapy, which often includes regular hemodialysis and peritoneal dialysis [6]. Among these, maintenance hemodialysis (MHD) is widely used and is a relatively mature form of renal replacement therapy. However, dialysis treatment is long-term and time-sensitive. During this treatment, patients are more prone to depression, anxiety, and a series of psychological disorders than the general population [7].

Depression is the most common mental disorder in maintenance hemodialysis patients [8]. It decreases the quality of life for these patients and has a significant negative impact on their clinical course and prognosis. Studies have indicated that 25%–60% of MHD patients experience varying degrees of depressive symptoms [9, 10]. Depression can manifest in many forms and intensities, both mental and physical, ranging from mild sadness to severe pain or even suicidal tendencies [11]. As the duration of dialysis extends in MHD patients, the incidence of complications increases, quality of life decreases, economic pressure rises, and the degree of depression also intensifies [12]. It has been demonstrated that there is a significant correlation between depression and mortality in maintenance dialysis patients [13].

Some studies have shown that psychological interventions, such as psychosocial interventions, positive psychological interventions, and cognitive behavioral therapy, can help improve depression in hemodialysis patients [14, 15, 16]. Psychiatric Association guidelines also highlight the therapeutic effects of psychological interventions in patients with depression and anxiety. Psychological interventions can improve depression, anxiety, and the quality of life of MHD patients to a certain extent. However, research conclusions are not uniform, and the sample sizes of some studies are small [17]. In recent years, with the deepening of research on psychological interventions to improve the psychological state and quality of life of MHD patients both domestically and internationally, the intervention effects have been further confirmed. Therefore, this study conducted a systematic review and meta-analysis of all completed studies to explore the effects of psychological interventions on depression and anxiety in hemodialysis patients.

## Methods

### Literature Search

The main and abstract checklist of PRISMA were completed (**Supplementary File 1**). The required literature was searched in the PubMed, Embase, Web of Science, ScienceDirect, and Cochrane Library databases. The search timeframe covered the period from the establishment of each database to October 2023. The search was supplemented by a retrospective examination of references included in the identified literature. The search terms included: psychological intervention, cognitive-behavioral therapy (CBT), psychosocial interventions, positive psychological intervention, depression, anxiety, hemodialysis treatment, maintenance hemodialysis, and MHD. The search strategy combined subject terms and free terms: “psychological intervention, depression/anxiety AND hemodialysis treatment/maintenance hemodialysis/MHD”, “cognitive-behavioral therapy/CBT, depression/anxiety AND hemodialysis treatment/maintenance hemodialysis/MHD”, “positive psychological intervention, depression/anxiety AND hemodialysis treatment/maintenance hemodialysis/MHD”.

### Inclusion and Exclusion Criteria

Inclusion criteria: (1) Type of study: randomized controlled trial (RCT); (2) Subjects: patients undergoing hemodialysis treatment and diagnosed with depression; (3) Interventions: psychological interventions were used in all intervention groups. All psychological interventions aimed at treating depressive symptoms were included in the study.

Exclusion criteria: (1) Review, conference abstracts; (2) Duplicate publication; (3) Full text not available; (4) Non-English literature; (5) Incomplete data.

### Literature Quality Assessment and Information Extraction

The quality of the included literature was independently evaluated by two investigators according to the evaluation criteria recommended by the Cochrane Handbook for Systematic Evaluation [18]. In cases of disagreement, a third researcher was consulted or a joint discussion was held. Included studies were considered to be at low risk of bias if they met all the evaluation standards, at moderate risk of bias if they met some of the standards, and at high risk of bias if they did not meet any of the standards.

The following information was extracted from all included studies:

(1) Basic data of the literature, including the first author and publication year.

(2) Basic information of the study subjects, including sample size, gender, and age of the patients.

(3) Interventions: intervention measures, intervention frequency, and intervention duration in the intervention group and the control group.

(4) Outcome indicators.

(5) Potential biases.

### Statistical Methods

The meta-analysis was conducted using RevMan 5.4 software (The Nordic Cochrane Centre, Copenhagen, Denmark). Count data were presented as odds ratios (OR) with corresponding 95% confidence intervals (CI). Statistical heterogeneity among the included studies was assessed using the *p* value and I^2^ statistic. A *p* value greater than 0.1 and an I^2^ value less than 50% suggested no significant heterogeneity among the studies, leading to the adoption of a fixed-effects model for meta-analysis. Conversely, if the *p* value was equal to or less than 0.1, or the I^2^ value was 50% or higher, indicating significant heterogeneity, further analysis was conducted to identify the source of heterogeneity. In such cases, a random-effects model was utilized for meta-analysis. Sensitivity analysis was performed to explore the source of heterogeneity by altering the effect model and sequentially excluding each included study.

## Results

### Results of Literature Search

**Fig. 1.**
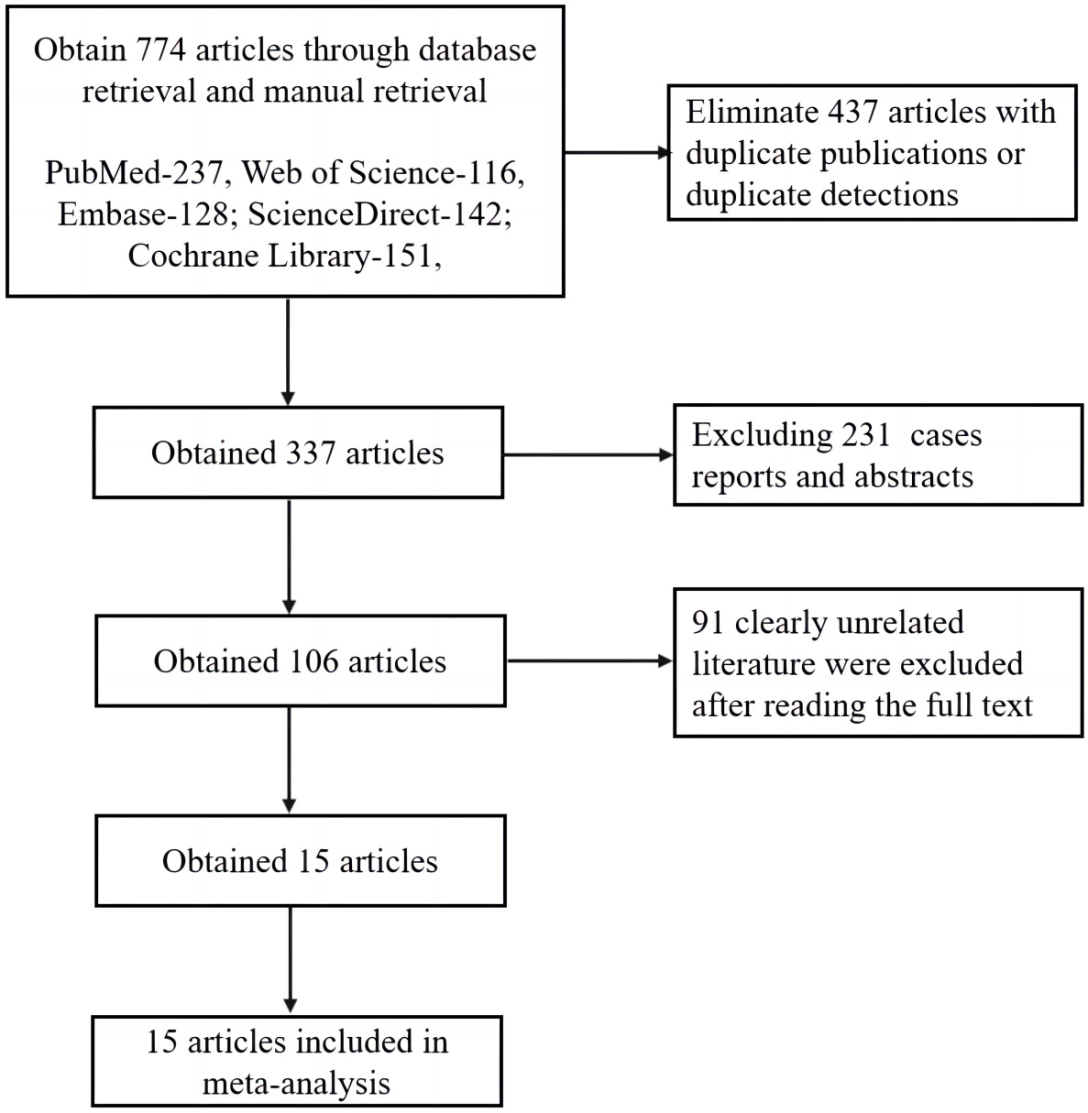
**Literature retrieval process**.

A preliminary search yielded 774 literature sources. Following primary and secondary screenings, duplicates, irrelevant articles, and studies lacking data were excluded. Ultimately, 15 studies were included in the meta-analysis (Fig. 1).

### Literature Quality Bias Evaluation

**Fig. 2.**
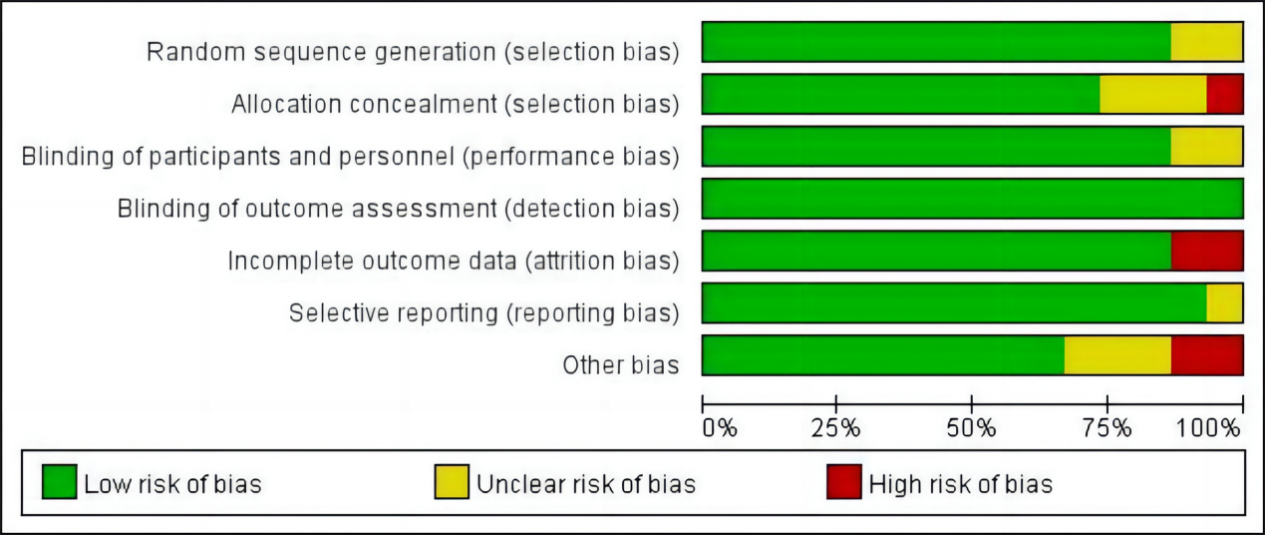
**Risk of bias graph**.

**Fig. 3.**
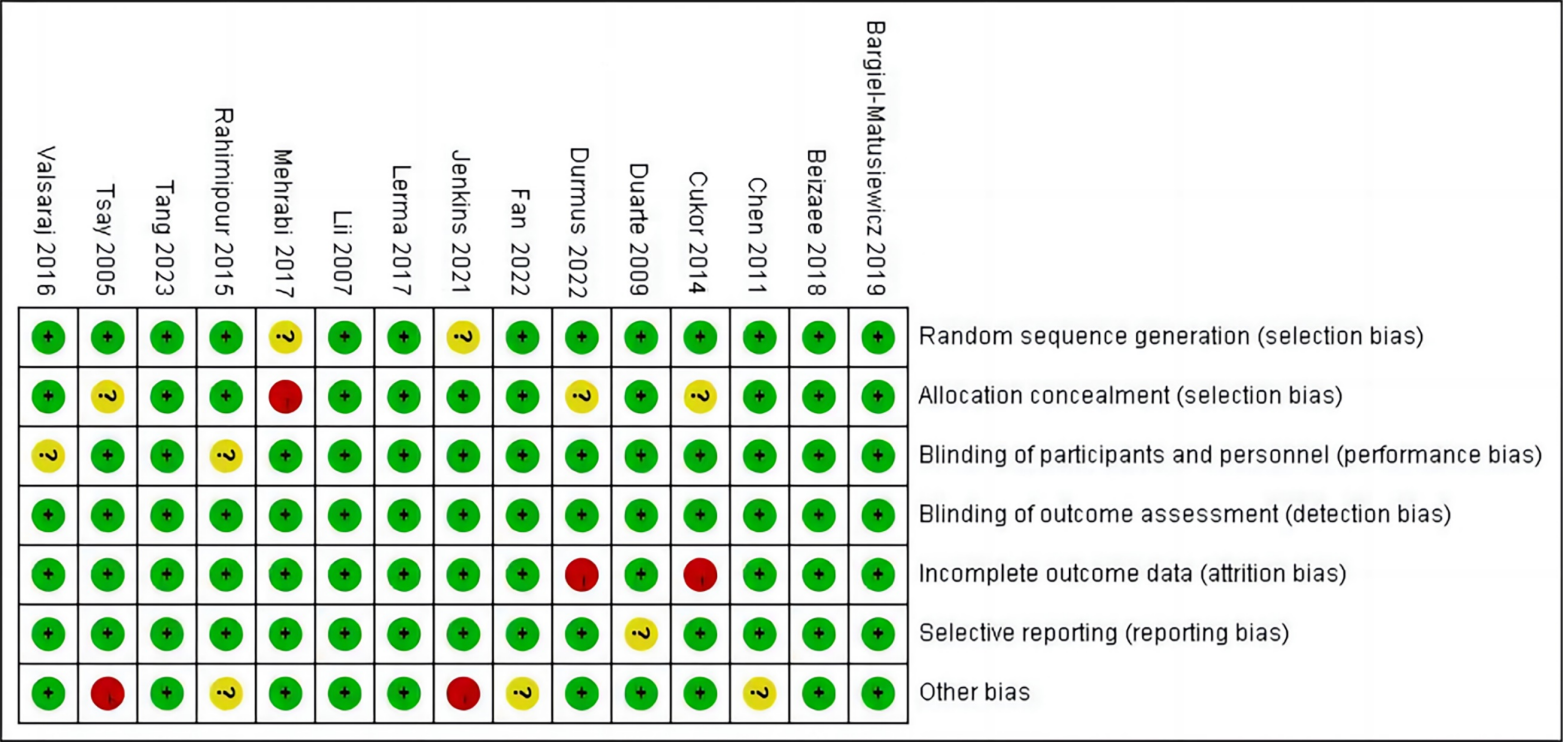
**Risk of bias summary**.

In terms of detection bias, all studies performed well with low risk of bias in detection bias, but there was a certain degree of ambiguity or high risk of bias in other aspects such as attrition bias, selection bias, performance bias, and other biases (Figs. 2,3).

### Basic Characteristics of the Included Literature

A total of 929 patients were enrolled, with 468 allocated to the intervention group and 461 to the control group. The intervention duration ranged from 4 to 12 weeks. Table 1 (Ref. [19, 20, 21, 22, 23, 24, 25, 26, 27, 28, 29, 30, 31, 32, 33]) presents the basic information of the included studies.

**Table 1.** **General characteristics of the included literature**.

Author	Year	Group	Age	Male/Female	Frequency	Intervening measure	Total duration	Outcome indicator
Bargiel-Matusiewicz, *et al*. [19]	2019	I = 45	48.44 ± 5.60	22/23	2/wk	Psychoeducation cognitive/narrative	4 wk	BDI/STAI
		C = 48	49.56 ± 9.94	26/22		Standard medical procedure		
Beizaee, *et al*. [20]	2018	I = 40	47.20 ± 8.36	24/16	3/wk	Guided imagery	4 wk	HADS
		C = 40	47.22 ± 5.43	23/17		Routine care		
Chen, *et al*. [21]	2011	I = 37	57 ± 9	17/20	3/wk	Cognitive-behavioral therapy	6 wk	BDI/BAI
		C = 35	59 ± 11	13/22		Sleep hygiene education		
Cukor, *et al*. [22]	2014	I = 33	-	6/27	-	Cognitive-behavioral therapy	12 wk	BDI
		C = 26	-	6/20		Routine care		
Duarte, *et al*. [23]	2009	I = 41	52.4 ± 15.9	15/16	-	Cognitive-behavioral therapy	12 wk	BDI
		C = 44	54.0 ± 12.7	20/22		Routine care		
Durmuş, *et al*. [24]	2022	I = 33	-	23/10	2/wk	Spiritual care	8 wk	HADS
		C = 38	-	20/18		Standard treatment		
Fan, *et al*. [25]	2022	I = 30	52.01 ± 4.81	18/12	-	Psychological intervention	4 wk	SAS/SDS
		C = 30	52.14 ± 5.25	17/13		Routine interventions		
Jenkins, *et al*. [26]	2021	I = 13	60.8 ± 10.19	-	1/wk	Individualized support	9 wk	HADS
		C = 17	59.78 ± 13.19	-		Routine care		
Lerma, *et al*. [27]	2017	I = 31	41.8 ± 14.7	15/16	5/wk	Cognitive-behavioral therapy	5 wk	BDI/BAI
		C = 18	41.7 ± 15.1	8/10		Standard care		
Lii, *et al*. [28]	2007	I = 20	58.2 ± 10.9	10/20	1/wk	Cognitive-behavioral therapy	8 wk	BDI
		C = 28	59.0 ± 11.4	13/15		Routine care		
Mehrabi, *et al*. [29]	2017	I = 25	-	-	1/wk	Happiness training	6 wk	BDI/SAS
		C = 25	-	-		Routine care		
Rahimipour, *et al*. [30]	2015	I = 25	-	-	1/wk	Hope therapy	8 wk	Depression anxiety
		C = 25	-	-		Routine care		
Tang, *et al*. [31]	2023	I = 32	64.25 ± 5.68	17/15	1/wk	Psychological care	12 wk	HADS
		C = 26	67.65 ± 7.70	14/12		Routine care		
Tsay, *et al*. [32]	2005	I = 30	-	14/16	1/wk	Adaptation training	8 wk	BDI
		C = 27	-	13/14		Routine care		
Valsaraj, *et al*. [33]	2016	I = 33	-	23/10	1/wk	Cognitive-behavioral therapy	8 wk	HADS
		C = 34	-	24/10		Routine care		

Note: C, control group; I, intervention group; BDI, Beck Depression Inventory; BAI, Beck Anxiety Inventory; HADS, Hospital Anxiety and Depression Scale; SDS, Self-rating depression scale; SAS, Self-rating anxiety scale; STAI, State-Trait Anxiety Inventory.

### Meta Analysis of Depression

**Fig. 4.**
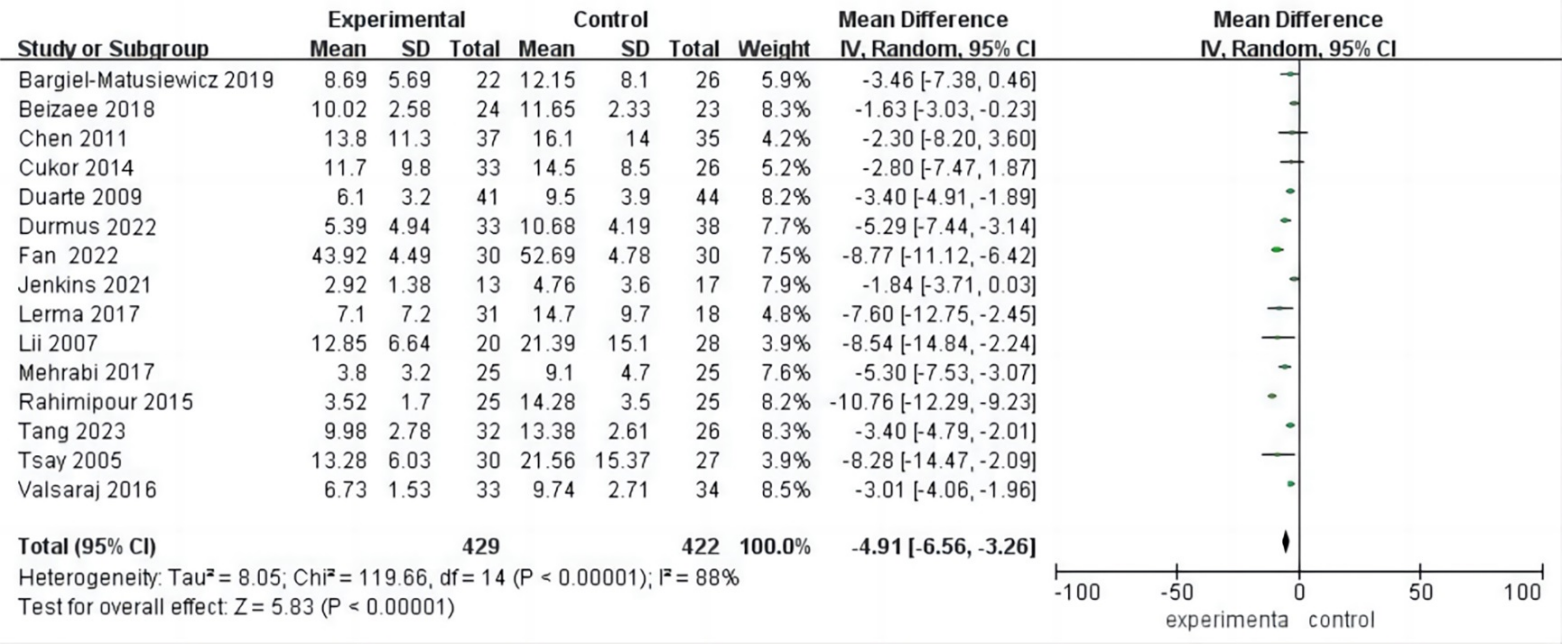
**Forest plot of depression**. CI, confidence intervals.

All 15 included studies provided comprehensive data on post-intervention depression. With an I^2^ of 88% and a *p* value of less than 0.00001, indicating high heterogeneity, a random-effects model was employed. The analysis revealed a statistically significant improvement in depression following psychological intervention compared to the control group [mean difference (MD) = –4.91, 95% CI (–6.56, –3.26), *p* < 0.001]. These findings suggest the effectiveness of psychological intervention in reducing depression levels among hemodialysis patients (Fig. 4).

### Subgroup Analysis of Depression

**Fig. 5.**
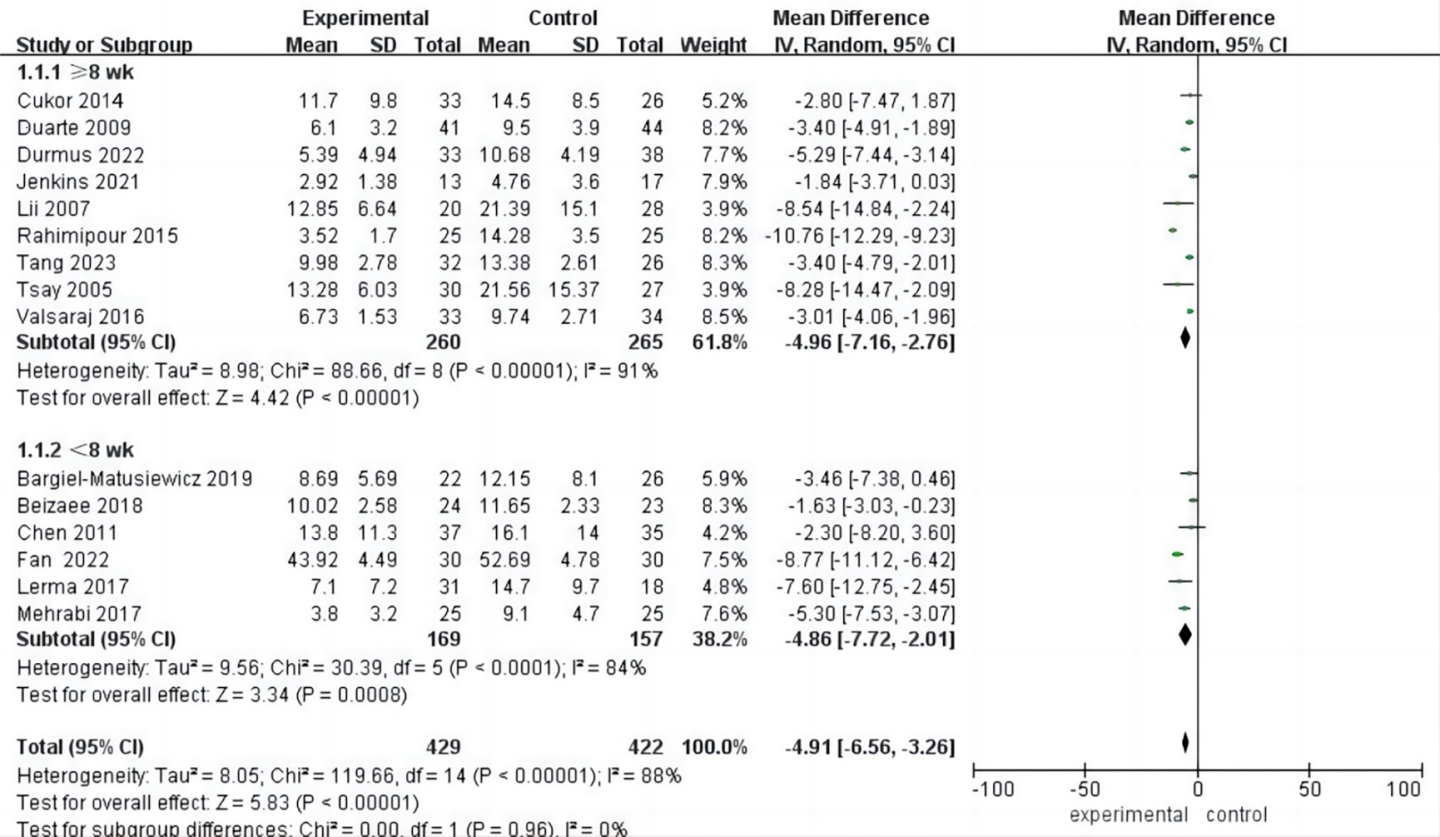
**Forest plot of subgroup analysis of intervention duration for depression**.

Among the included studies, nine provided data on psychological interventions lasting ≥8 weeks, while the remaining six reported interventions of less than 8 weeks. In both subgroups, there was a statistically significant improvement in depression. For interventions ≥8 weeks, the MD was –4.96 (95% CI (–7.16, –2.76), *p* < 0.001), with high heterogeneity (I^2^ = 91%, *p* < 0.001). Similarly, for interventions <8 weeks, the MD was –4.86 (95% CI (–7.72, –2.01), *p* < 0.001), also with high heterogeneity (I^2^ = 84%, *p* < 0.001). Notably, there was no significant difference in the improvement of depression between the two duration subgroups (*p* = 0.96) (Fig. 5).

### Meta Analysis of Anxiety

**Fig. 6.**
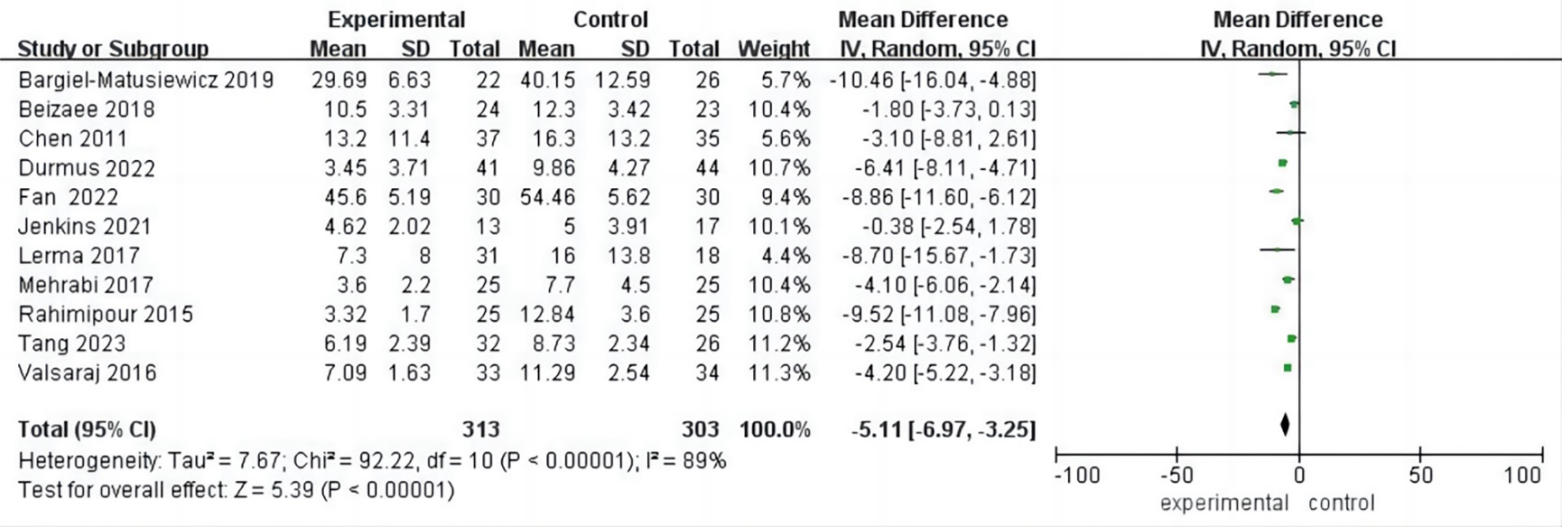
**Forest plot of anxiety**.

Among the 11 included studies, detailed data on post-intervention anxiety were provided. With an I^2^ of 89%, indicating high heterogeneity, the analysis showed a significant improvement in anxiety following psychological intervention compared to the control group (MD = –5.11, 95% CI (–6.97, –3.25), *p* < 0.001). These findings suggest that psychological intervention is effective in reducing anxiety levels among hemodialysis patients (Fig. 6).

### Subgroup Analysis of Anxiety

**Fig. 7.**
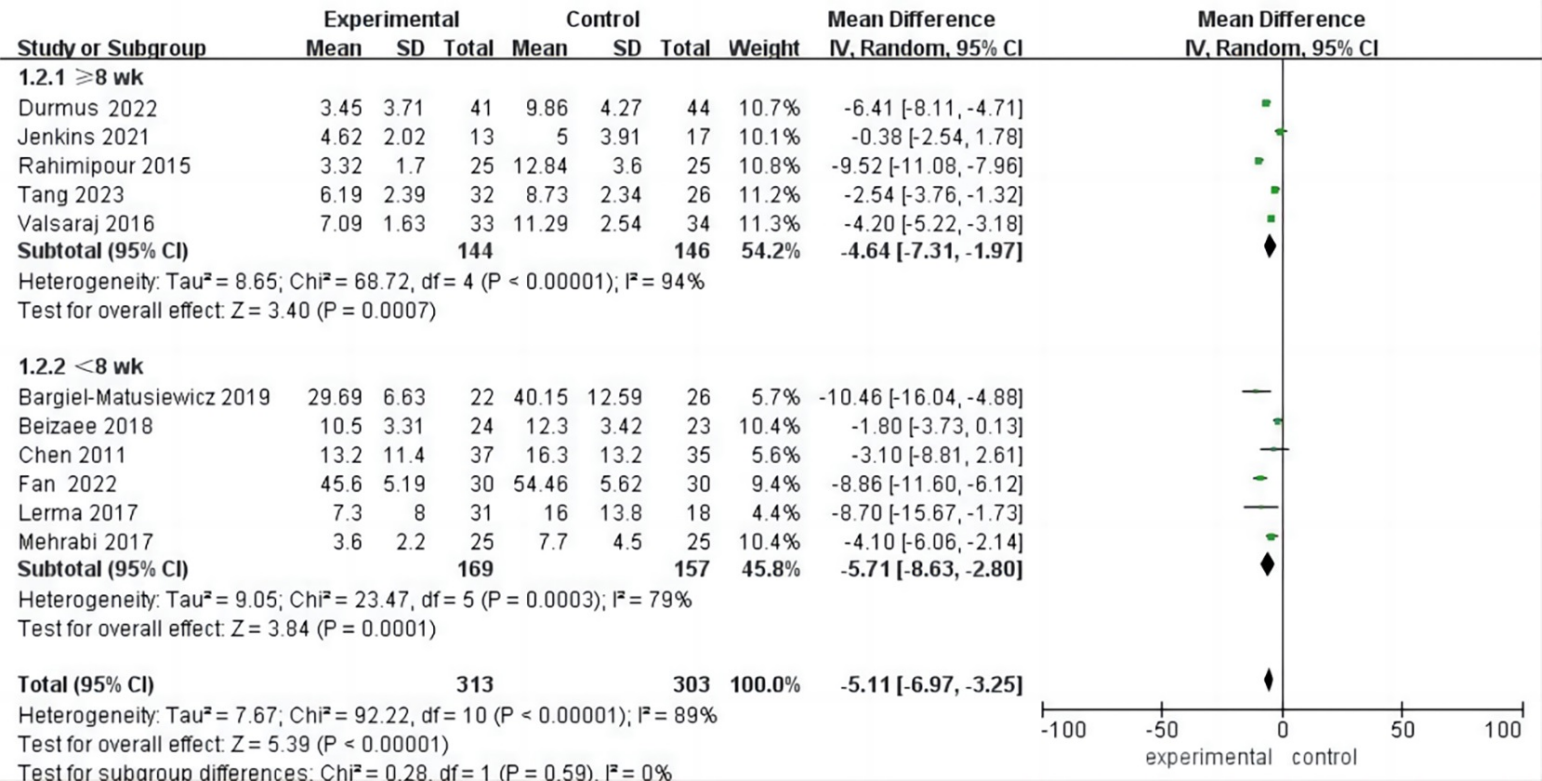
**Forest plot of subgroup analysis of intervention duration for anxiety**.

Five studies [24, 26, 30, 31, 33] provided anxiety data for psychological interventions lasting ≥8 weeks, while six studies [19, 20, 21, 25, 27, 29] reported data for interventions lasting less than 8 weeks. In the subgroup with interventions ≥8 weeks, anxiety showed a statistically significant improvement (MD = –4.64, 95% CI (–7.31, –1.97), *p* < 0.001), with high heterogeneity (I^2^ = 94%, *p* < 0.001). Similarly, in the subgroup with interventions lasting less than 8 weeks, there was also a statistically significant improvement in anxiety (MD = –5.11, 95% CI (–6.97, –3.25), *p* < 0.001), with high heterogeneity (I^2^ = 89%, *p* < 0.001) (Fig. 7).

### Subgroup Analysis of Life Quality

**Fig. 8.**
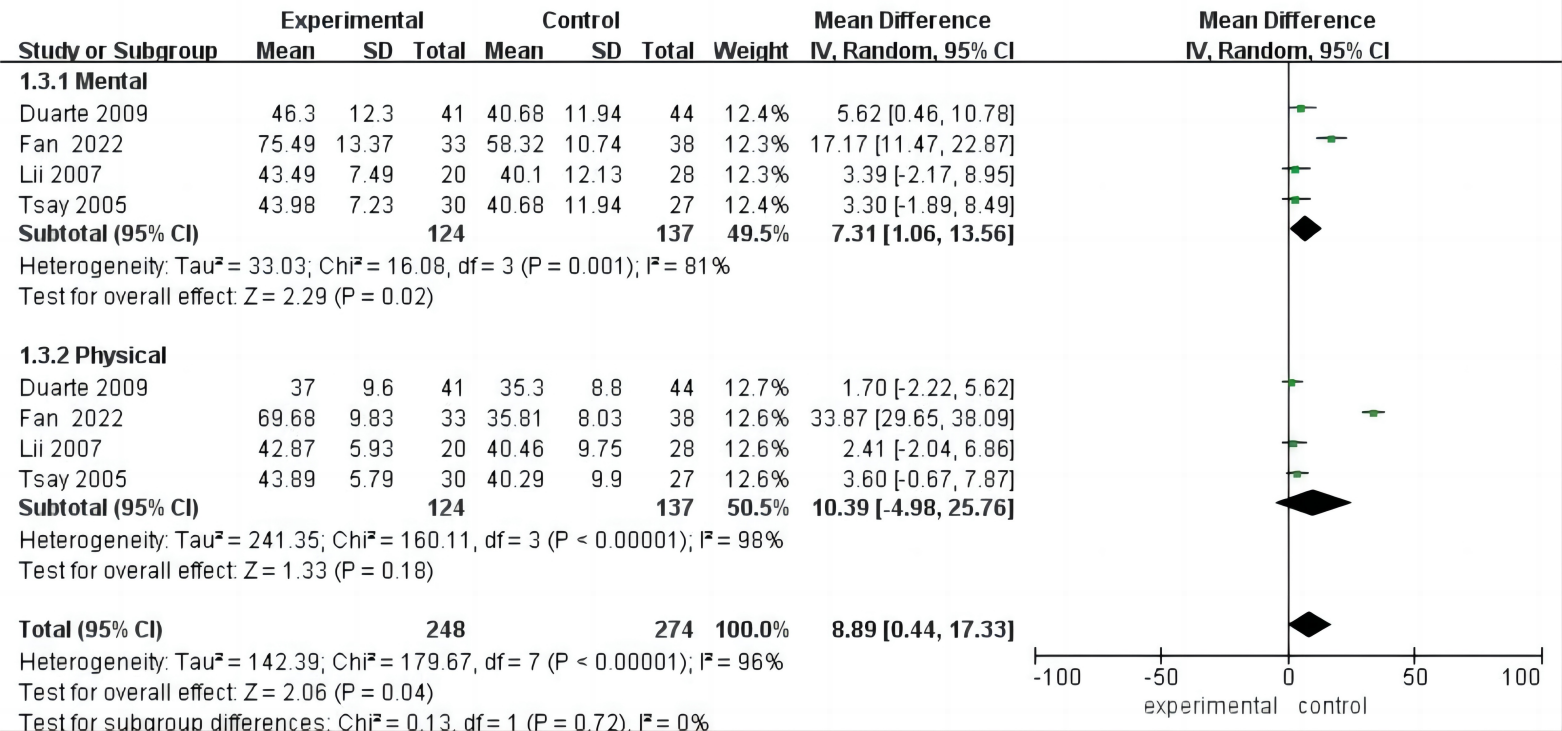
**Forest plot of subgroup analysis of life quality**.

Four studies [23, 25, 28, 32] reported both physical and psychological patient scores, prompting subgroup analyses to assess whether psychological interventions could enhance physical or psychological quality of life. The analysis revealed a statistically significant improvement in psychological functioning within the intervention group compared to the control group (MD = 7.31, 95% CI (1.06, 13.56), *p* = 0.001), albeit with heterogeneity across studies (I^2^ = 81%, *p* < 0.001). Conversely, there was no statistically significant improvement in physical functioning compared to the control group (MD = 10.39, 95% CI (–4.98, 25.76), *p* = 0.18), with heterogeneity also observed among studies. These findings underscore a significant effect of psychological interventions on the psychological aspects of quality of life. Furthermore, no substantial differences were observed between the two subgroups (*p* = 0.72) (Fig. 8).

### Sensitivity Analysis

During sensitivity analysis, it was observed that Rahimipour’s study [30] contributed to heterogeneity in the effect of psychological interventions lasting ≥8 weeks on depression. Upon its exclusion, interventions of this duration continued to demonstrate a statistically significant improvement in depression, with the I^2^ decreasing to 37%. Similarly, Fan’s study [25] was identified as a source of heterogeneity in the subgroup analysis of quality of life. Following its exclusion, psychological interventions exhibited a significant effect on both psychological MD = 4.14, 95% CI (1.08, 7.20), *p* = 0.008) and physical (MD = 2.52, 95% CI (0.10, 4.95), *p* = 0.04) aspects of quality of life among hemodialysis patients, with reduced heterogeneity (I^2^ = 0%).

## Discussion

In this study, we included 15 relevant literatures and conducted a meta-analysis involving 929 patients undergoing MHD with depression. Our findings indicate that psychological interventions contribute to the amelioration of depression in MHD patients with MHD, aligning with the conclusions drawn in Zegarow systematic review [34]. It’s noteworthy that individuals experiencing depressive states often manifest cognitive distortions, characterized by exaggerated or irrational thought patterns that foster a negative perception of reality [35].

Psychological intervention stands out as a pivotal approach for fostering the reconfiguration of negative thought patterns, managing emotional states, and facilitating behavioral adjustments, making it the most effective and frequently utilized psychological treatment in alleviating anxiety and depression among hemodialysis patients [36]. At its core, psychological intervention aims to elucidate the interplay between patients’ cognition, emotions and adaptive behaviors, aiding them in cultivating accurate cognitive frameworks that foster adaptive behaviors. After psychological intervention, dialysis patients showed significant improvement in depression, and their quality of life and adherence to treatment were also improved. Ng’s study [37] included 8 papers for meta-analysis of the results of psychological intervention treatment for MHD comorbid with depression, and psychological intervention was able to improve the patients’ depression, anxiety and enhance the patients’ quality of life. Similarly, another meta-analysis involving 9 studies highlighted the potential promise of psychological treatment in managing depression among dialysis patients, with significant improvements observed post-treatment [38]. These are consistent with the results of our study, reinforcing the beneficial effects of psychological intervention in this patient population.

Furthermore, the efficacy of psychological intervention in alleviating anxiety among MHD patients is evident. Studies have indicated that a duration of 8 weeks for psychological intervention can notably decrease anxiety levels and enhance treatment response rate [39]. Our study corroborates these findings, demonstrating significant improvements in anxiety status among MHD patients irrespective of whether the duration of psychological intervention exceeded 8 weeks. This further underscores the benefits of psychological intervention in this patient population.

Regarding quality of life, the results of a meta-analysis based on 4 Randomized Controlled Trials (RCTs) revealed that psychological interventions contributed to improvements in the psychological aspects of patients’ quality of life, albeit without a significant effect on the physical aspects. However, upon exclusion of sources of heterogeneity, psychological interventions exhibited significant improvements in both psychological and physiological aspects of patients’ quality of life uniformly. This discrepancy may be attributed to the limited number of included studies and variations in evaluation indicators. Therefore, it underscores the necessity for further validation of the impact of psychological interventions on patient quality of life. Future RCTs should incorporate quality of life assessments to comprehensively evaluate the extent of psychological interventions’ impact on patients.

Despite encountering high heterogeneity in certain indicators, this study’s reliability remains intact following sensitivity and subgroup analyses, which effectively mitigated heterogeneity without altering the original statistical outcomes. However, it’s crucial to acknowledge certain limitations. The study exclusively included English literature, potentially introducing publication bias by overlooking other languages and grey literature. Moreover, only a portion of the included studies utilized CONSORT flow charts to depict randomization processes and loss to follow-up, introducing selection bias. Furthermore, inconsistencies in outcome assessment tools, small sample sizes, vague descriptions of intervention measures, and lack of specificity regarding the qualifications of trainers contributed to heterogeneity. To address these limitations, future Randomized Controlled Trials (RCTs) should adhere to standardized study designs, utilize CONSORT guidelines for randomization and follow-up reporting, provide detailed descriptions of interventions, and ensure the professionalism and qualifications of trainers are transparently outlined. Additionally, employing psychological interventions as positive guides can enhance study fidelity.

## Conclusion

The findings from this meta-analysis involving 929 hemodialysis patients with comorbid depression underscore the potential therapeutic benefits of psychological interventions compared to conventional approaches. Particularly notable is the significant improvement observed in patients’ depression, anxiety, and quality of life following psychological interventions. Given the substantial psychological burden associated with hemodialysis, the implementation of such interventions holds promise for effectively addressing depression and enhancing mental health outcomes among these patients.

## Availability of Data and Materials

The datasets used and/or analysed during the current study were available from the corresponding author on reasonable request.

## Supplementary Material

Supplementary material associated with this article can be found, in the online version, at https://doi.org/10.62641/aep.v53i1.1628.
